# Digital Customized Titanium Mesh for Bone Regeneration of Vertical, Horizontal and Combined Defects: A Case Series

**DOI:** 10.3390/medicina57010060

**Published:** 2021-01-11

**Authors:** Daniele De Santis, Federico Gelpi, Giuseppe Verlato, Umberto Luciano, Lorena Torroni, Nadia Antonucci, Fabio Bernardello, Morris Zarantonello, Pier Francesco Nocini

**Affiliations:** 1 Department of Surgery, Dentistry, Paediatrics and Gynecology, University of Verona, 37134 Verona, Italy; daniele.desantis@univr.it (D.D.S.); umbe1@hotmail.it (U.L.); pierfrancesco.nocini@univr.it (P.F.N.); 2 Department of Diagnostics and Public Health, University of Verona, 37134 Verona, Italy; giuseppe.verlato@univr.it (G.V.); lorena.torroni@univr.it (L.T.); nadia.antonucci@univr.it (N.A.); 3 Free Practitioner in Verona Via F.Bonvicini 42, Legnago, 37045 Verona, Italy; fabio.tredici@libero.it; 4 Free Practitioner in Verona Via Carducci 88/2 Badia Polesine, Rovigo, 45021 Verona, Italy; morris.zarantonello@hotmail.it

**Keywords:** custom titanium mesh, bone regeneration, digital planned surgery

## Abstract

*Background and Objective:* Guided bone regeneration allows new bone formation in anatomical sites showing defects preventing implant rehabilitation. *Material and Methods:* The present case series reported the outcomes of five patients treated with customized titanium meshes manufactured with a digital workflow for achieving bone regeneration at future implant sites. A significant gain in both width and thickness was achieved for all patients. *Results*: From a radiographic point of view (CBTC), satisfactory results were reached both in horizontal and vertical defects. An average horizontal gain of 3.6 ± 0.8 mm and a vertical gain of 5.2 ± 1.1 mm. *Conclusions:* The findings from this study suggest that customized titanium meshes represent a valid method to pursue guided bone regeneration in horizontal, vertical or combined defects. Particular attention must be paid by the surgeon in the packaging of the flap according to a correct method called the “poncho” technique in order to reduce the most frequent complication that is the exposure of the mesh even if a partial exposure of one mesh does not compromise the final outcome of both the reconstruction and the healing of the implants.

## 1. Introduction

Implant dentistry has become a very reliable method for restoring aesthetics and function in totally or partially edentulous patients [1]. However, tooth loss inevitably induces alveolar bone resorption with a resulting horizontal, vertical or combined volumetric defect.

The main goal of reconstructive surgery is to exploit the regenerative potential of the native bone in order to achieve new bone formation allowing an easier implant rehabilitation [2]. Guided bone regeneration (GBR) is a particular surgical procedure that allows new bone formation in anatomical sites showing vertical, horizontal or combined defects or atrophies. The first principle of GBR is to place a mechanical barrier to protect the blood clot, isolating the defect from the outlining connective tissue.

Titanium-reinforced polytetrafluoroethylene (PTFE) membranes stabilized with pins and screws are often considered the most predictable tools for increasing bone volume in large alveolar ridge deficiencies prior to or during implant surgery [3]. There is a huge heterogeneity of scientific data and opinions concerning which membrane, non-absorbable or resorbable, would be the most suitable [4].

In 2006, Wang described four steps to achieve success with the GBR technique. The author used the acronym “PASS” [5]: primary closure, angiogenesis, stability and space creation. To meet all of the above-mentioned criteria, it is necessary to use a containing and resistant structure (stability, space creation) filled with particulate bone (which promotes an ideal angiogenesis) followed by an accurate management of soft tissues (primary closure) [6]. The implant success rate related to GBR techniques has been reported to be between 68% and 100% [7,8].

**Figure 1:**
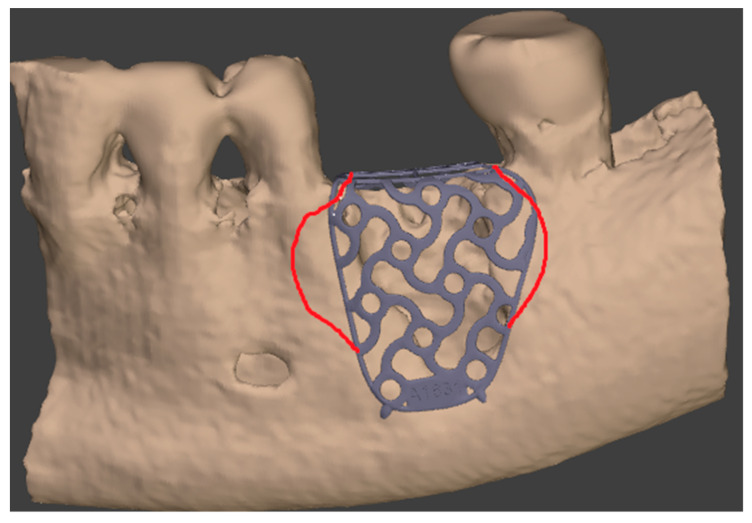
The digital planning of the custom made titanium mesh with the feedback corrections.

Titanium mesh has an excellent biocompatibility thus avoiding intolerance problems. The formation of a layer of TiO2 (titanium dioxide) on the surface seems to stimulate the osteo-genetic activity of osteoblasts [9]. New advances in tissue engineering technology such as computer-aided design (CAD) and computer-aided manufacturing (CAM) have significantly improved the clinical performance of those barriers [9,10,11,12]. A significant benefit of titanium membranes is the possibility to prepare customized devices for bone augmentation, individually suited for patients requiring implant rehabilitation [13,14]. The rigid fixation of the mesh to the bone with micro-screws gives an ideal stability throughout the healing period (Figure 1).

Furthermore, the presence of holes in the grid structure enhances a greater blood and cellular supply starting from the periosteum. The exploitation of osteo-genetic resources arising from the periosteum allows the success of the therapy even in association with non-autologous grafting materials. [15,16].

The purpose of the present case series study was to evaluate the efficacy of the bone augmentation procedure with the use of a customized titanium mesh both in vertical and horizontal defects.

## 2. Materials and Methods

The present study is a case series including patients that had been treated in the Maxillofacial Surgery and Dentistry Unit of the Hospital G.B. Rossi in Verona, Borgo Roma.

Inclusion criteria were:

- The presence, clinically and radiographically (intraoral radiographs, panoramic, CT scans) assessed, of horizontal, vertical or mixed bone defects of the maxillary bones in particular the presence of residual bone <8 mm in height and <5 mm in width.
- The absence of any local or systemic contraindication to surgical treatment such as infections, a smoking habit of >10 cigarettes a day, uncontrolled diabetes (HBA1c ≥ 7.5%), previous radiotherapy in the head and neck anatomical areas, chemotherapy, liver, blood and kidney diseases, immunosuppression, state of pregnancy, inflammatory and autoimmune diseases of the oral cavity, poor oral hygiene and poor motivation.
- The need for a staged treatment with the placement of dental implants eight months after bone regeneration.

Five partially edentulous patients were included in the present analysis: three out of five patients were treated in maxillary sites and two in mandibular sites. A total of 12 alveolar defects were regenerated. All surgeries were performed under local anesthesia.

For all patients, a mixture of autologous and heterologous bone (50:50 ratio) was used; the autologous graft was harvested from the mandibular branch whereas the heterologous graft was Creos Xenogain® by Nobel, a mineral matrix of deproteinized and delipidated bovine bone with preserved micro/macro structures with a ratio of calcium phosphate that reflected the composition of healthy human bone (data on file Nobel Biocare material properties of CXG/biomaterials and NIBEC).

**Figure 2:**
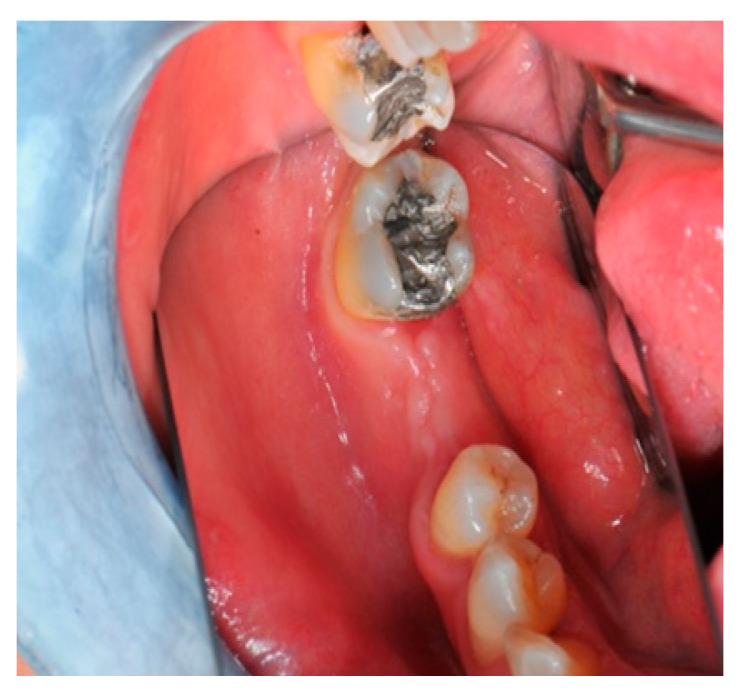
The occlusal aspect of the atrophic ridge before it is grafted.

**Figure 3:**
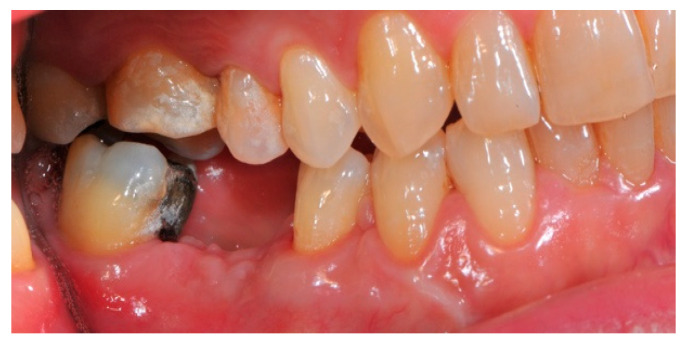
The initial buccal aspect of the atrophic mandible ridge before it is grafted.

**Figure 4:**
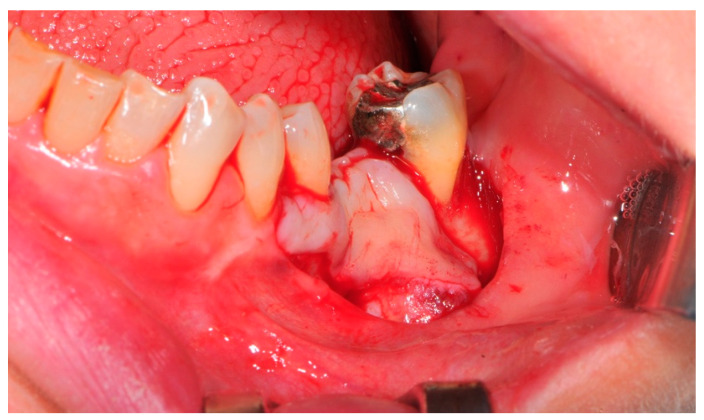
The bone defect exposure after the flap is raised up.

**Figure 5:**
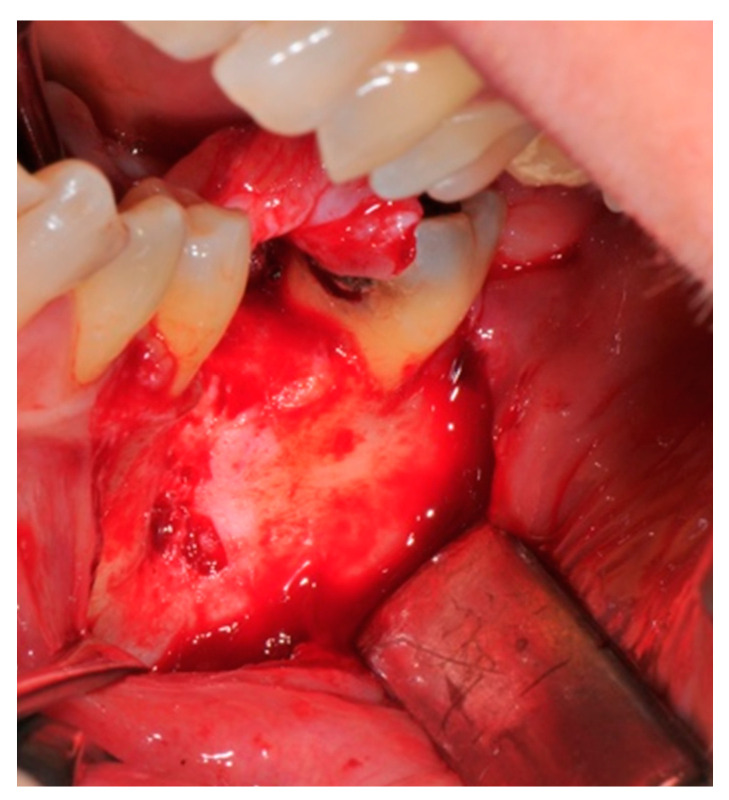
The design of the flap (“poncho” technique).

**Figure 6:**
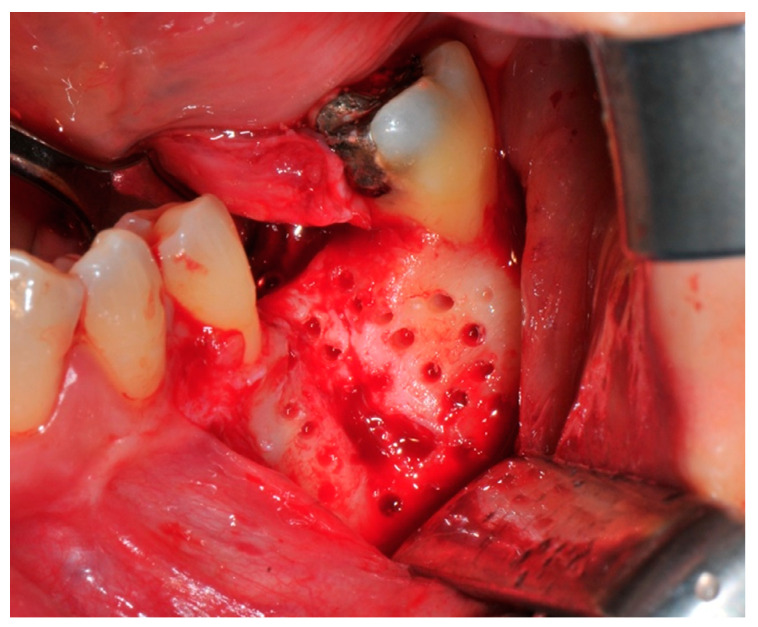
The receiving site before being grafted is cleaned and corticomized with multiple perforations.

**Figure 7:**
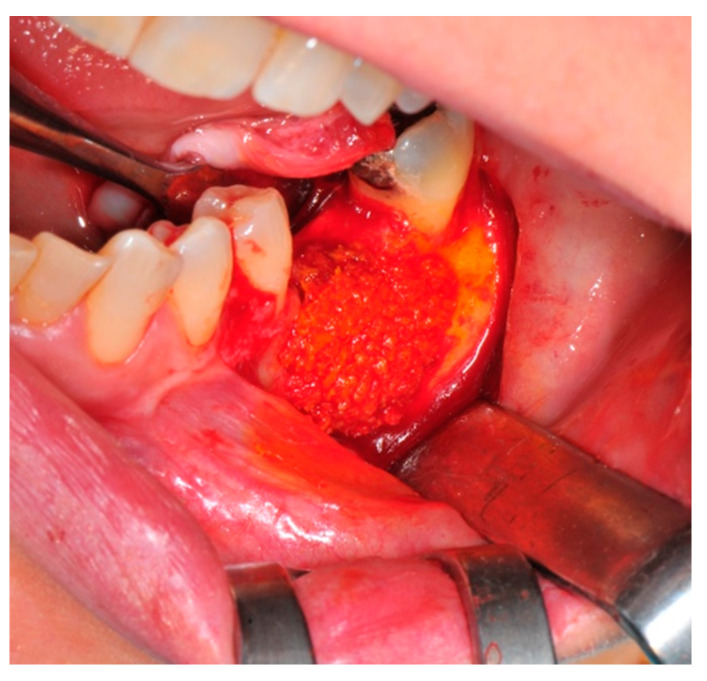
The bone graft in the correct position above the receiving site.

**Figure 8:**
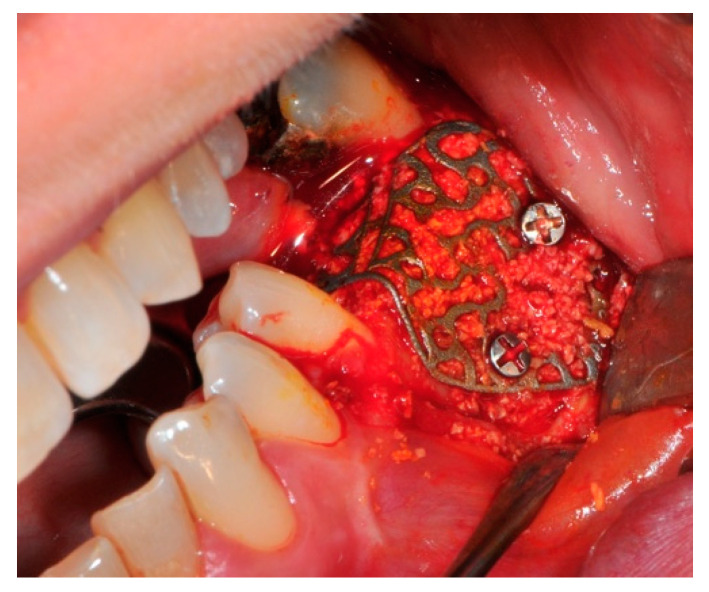
The titanium mesh in the correct position to prevent bone graft displacement.

**Figure 9:**
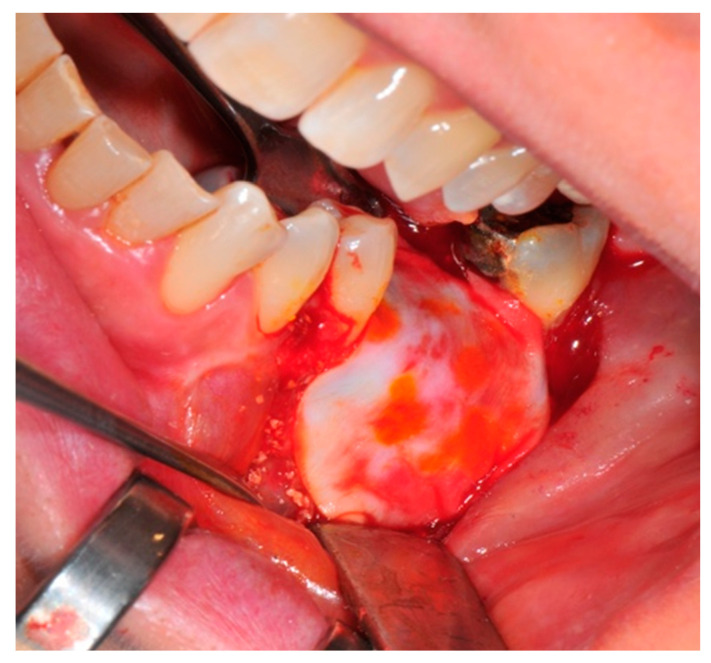
The resorbable membrane coverage above the titanium mesh.

**Figure 10:**
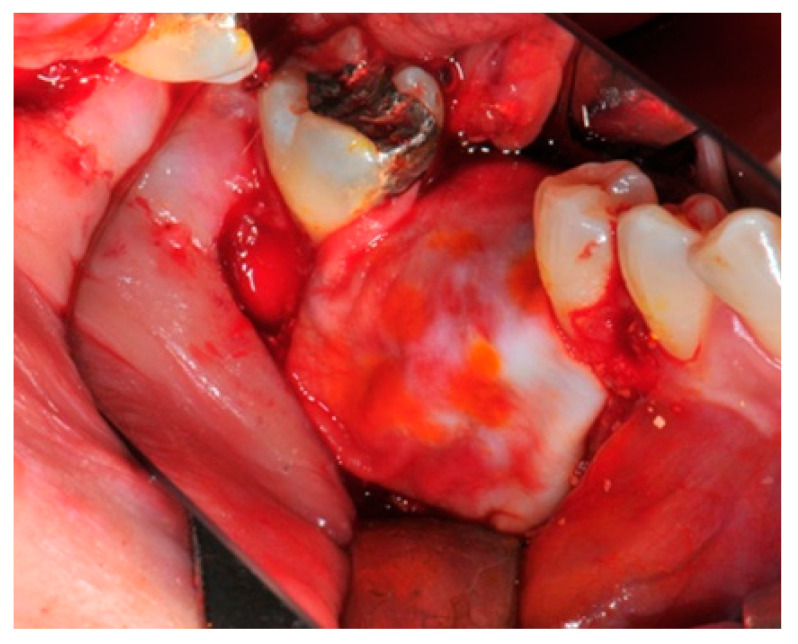
This is the final surgical step before the suture of the tension free flap.

**Figure 11:**
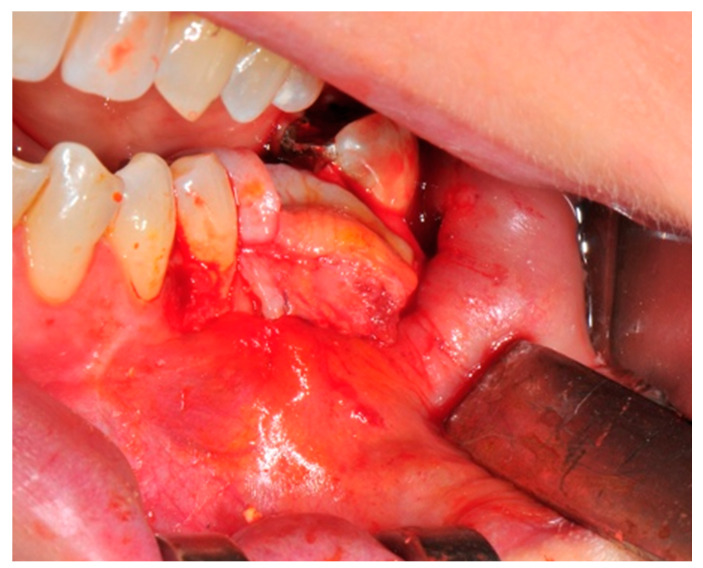
The flap must be tension-free.

**Figure 12:**
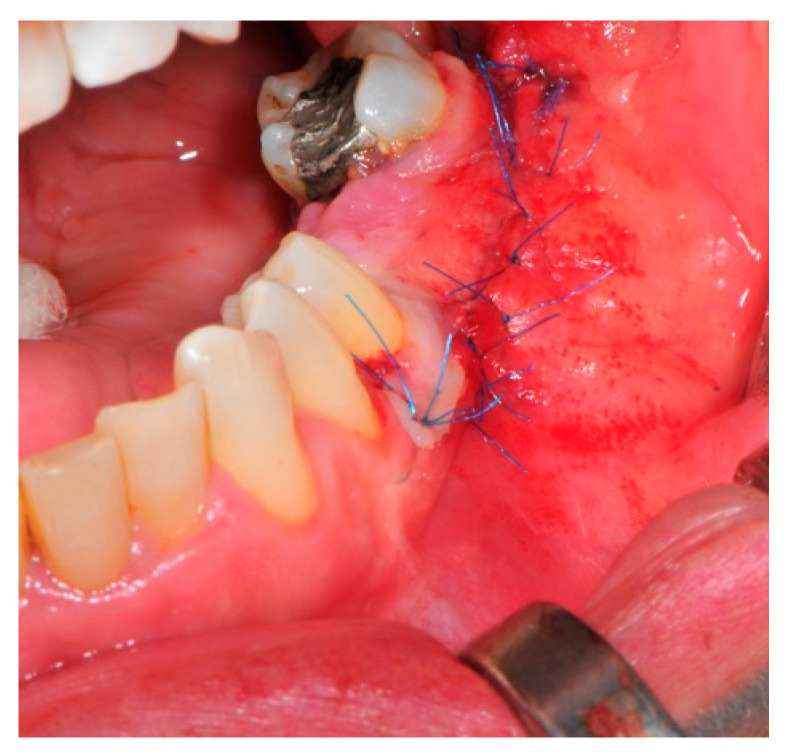
The buccal aspect of the sutured flap.

**Figure 13:**
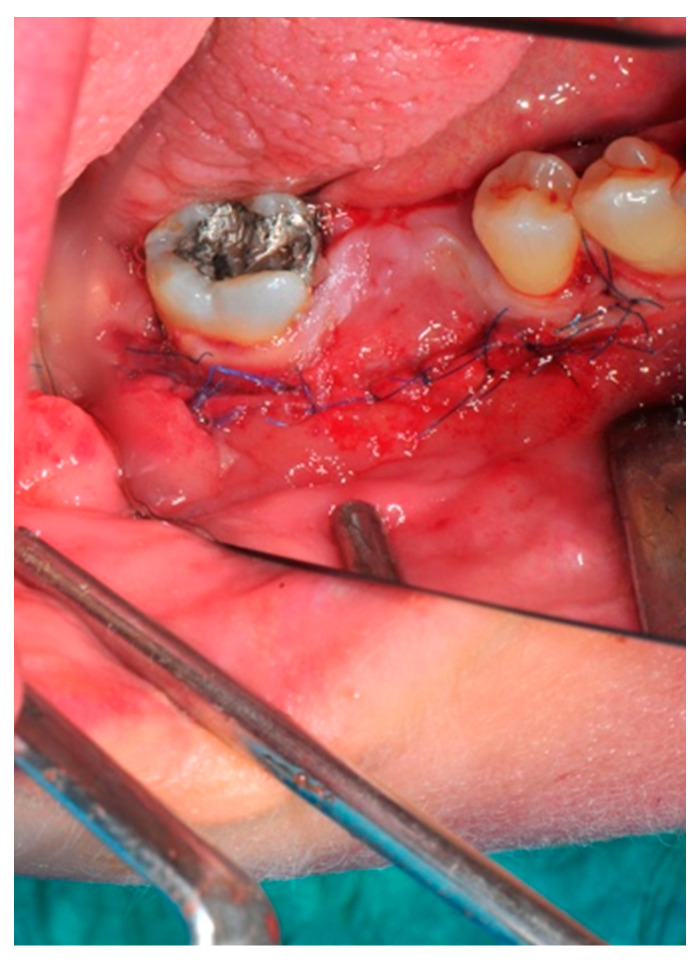
The primary closure of the poncho designed flap is well established.

The surgical operative protocol was carried out according to the following stages:

- Flap design: Soft tissue management should be as accurate as possible. The design of the flap should ensure a tension-free primary closure of the wound even after voluminous grafting of the defect. One option (preferred especially in wide vertical defects) is the execution of the so-called “poncho” flap. This technique includes a high vestibular incision of the mucosa, muscle and periosteum in order to undermine the preparation of the flap and to achieve its mobilization, followed by a deep incision in the buccal area with two additional vertical incisions that are performed at an appropriate distance from the occlusal area and the site of augmentation. After the incision, the preparation of a muco-periosteal flap and the remotion of scar tissue, a full thickness flap is raised until the bone defect is uncovered [17,18,19,20]. Finally, the positioning of the customized titanium mesh is passively tested to evaluate its fit intra-operatively (Figure 2, Figure 3, Figure 4 and Figure 5).
- Preparation of the receiving site: The exposed bone is cleaned from all of the remaining soft tissue and it is then prepared with multiple perforations using a small ball bur; this procedure is known as “bone refreshing” and it is performed to expose the cancellous portion of the residual bone, which shows a great osteo-genetic potential (Figure 6).
- Particulate bone: At this stage, a 50:50 autologous/heterologous bone mix is grafted both in the atrophic site and within the titanium mesh (Figure 7).
- Positioning of the customized titanium mesh: Being patient-customized or responding to the specific requirements of the patient in terms of planned bone augmentation, this mesh does not require any modification and it should perfectly fit the patient’s residual bone (Figure 8).
- Fixation and coverage: Fixation is a crucial aspect of the procedure as the stability of the graft must be maintained in order to ensure bone regeneration. The grid is fixed on the residual bone with titanium screws. Subsequently, the titanium mesh is covered with a resorbable membrane (Figure 9 and Figure 10).
- Passivation of the flap: Delicate periosteal incisions are performed to achieve mobility of the flap and allowing first intention closure, creating a passive/tension-free flap in order to avoid detrimental tension or graft exposure (Figure 11).
- Suture: Perfect closure is achieved with a first line of horizontal mattress sutures positioned 5 mm from the incision line and, subsequently, with single interrupted stitches connecting the edges of the flap. With this technique, the edges of the flap are reversed, putting the inner layers of the connective tissue in close contact with each other. The intimate connection between the layers of connective tissue forms a barrier that largely prevents the exposure of the membrane (Figure 12 and Figure 13).

Antibiotic therapy (Amoxicillin and Clavulanic acid 3 g per day for 5 days) was prescribed in association with the assumption of a non-steroidal analgesic (2 per day for 3 days).

**Figure 14:**
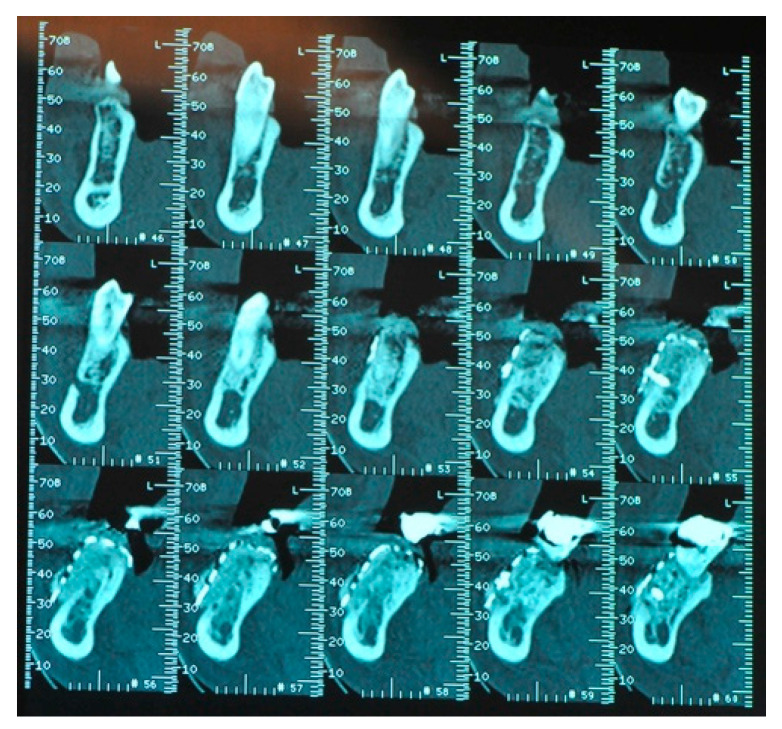
The CBCT ridge image after the bone graft healing period.

**Figure 15:**
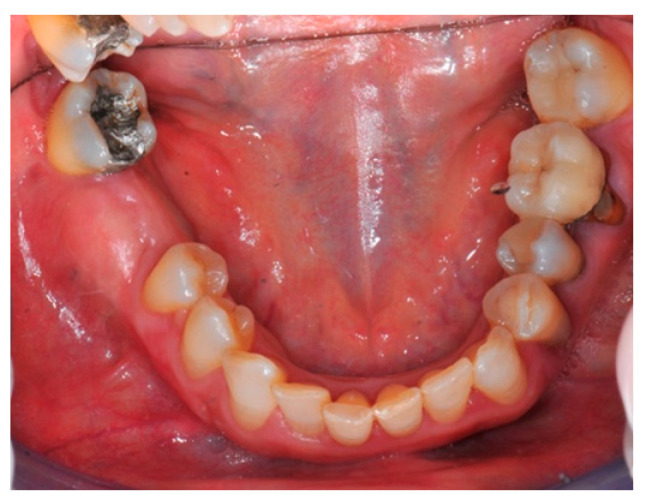
The clinical aspect of the same grafted area.

**Figure 16:**
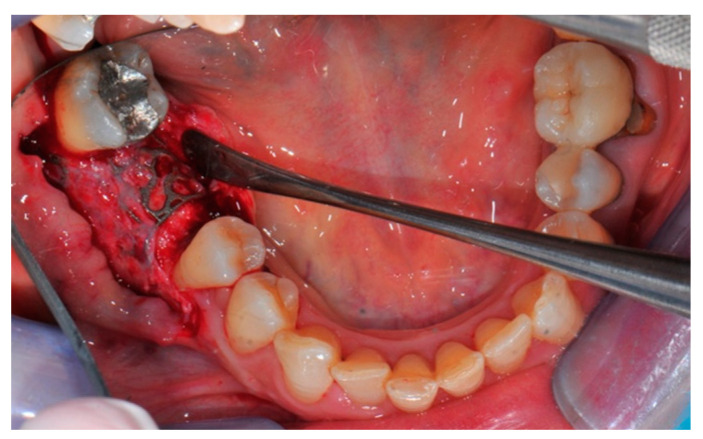
The titanium mesh removal.

**Figure 17:**
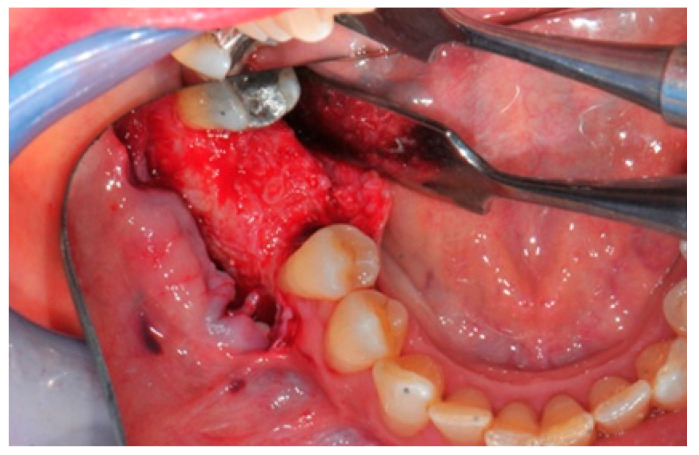
The new ridge after bone regeneration.

**Figure 18:**
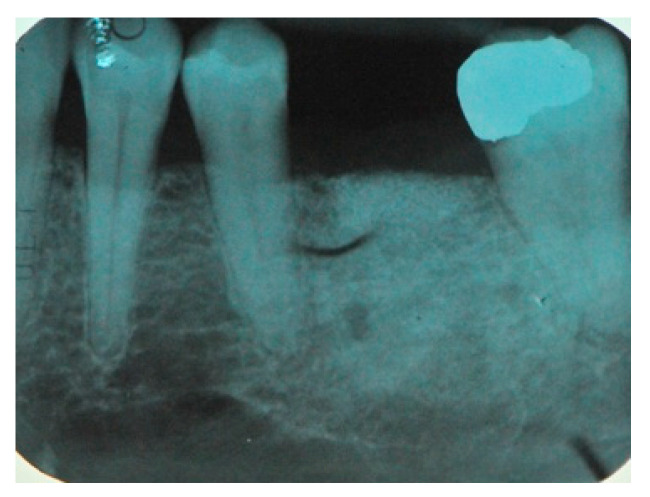
Radiological image of the new ridge before implant placement.

**Figure 19:**
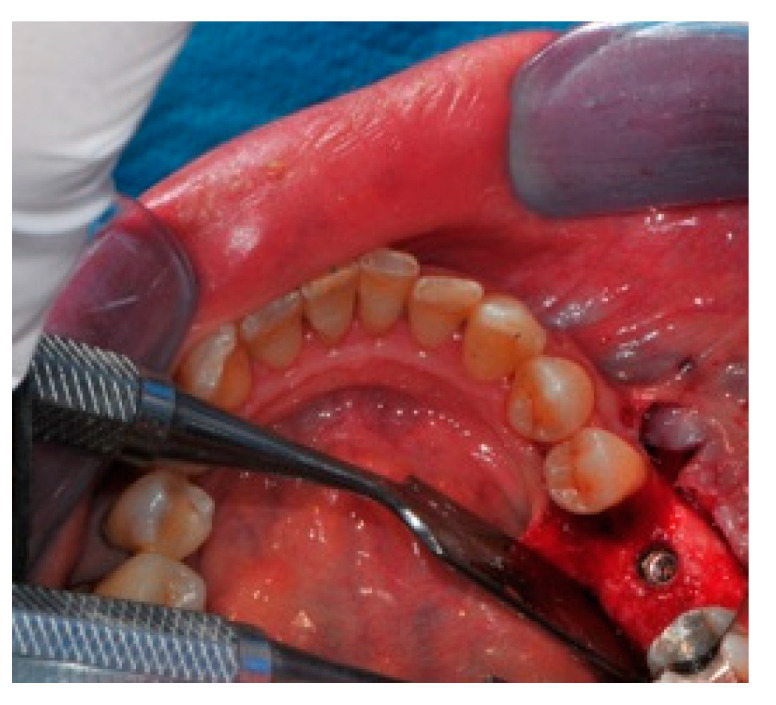
Occlusal aspect after implant placement.

**Figure 20:**
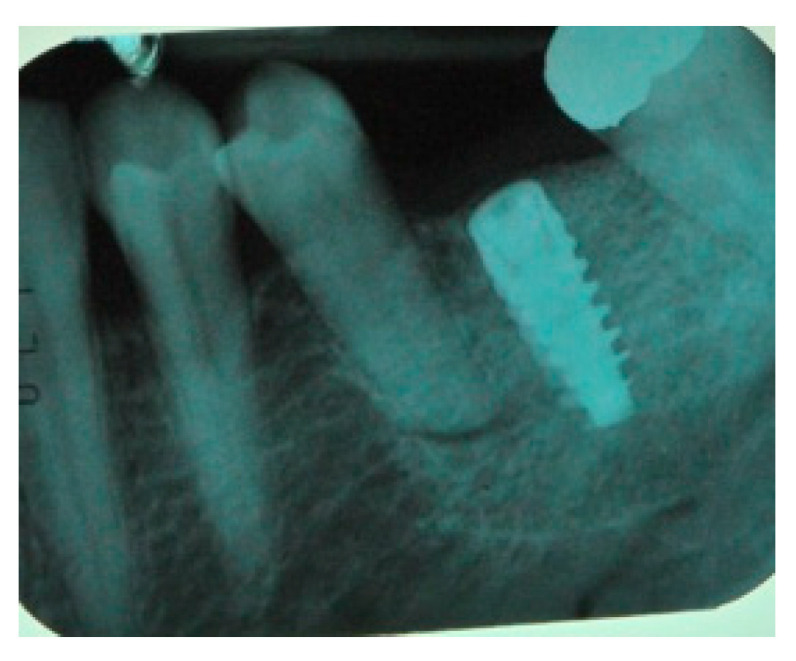
The radiological check after implant placement in the grafted area.

Eight months after surgery, patients were reviewed with a new CT scan in order to assess the quality and quantity of the new bone formation (Figure 14, Figure 15, Figure 16, Figure 17, Figure 18, Figure 19 and Figure 20).

This is the final surgical step of the dental restoration.

### Statistics

Descriptive and longitudinal statistics of the data were performed with R version 3.6.3 (29-02-2020)—“Holding the Windsock” (www.r-project.org/). A Brunner–Langer approach (mixed nonparametric method) was employed (Brunner and Puri 2001) was used to measure differences in the samples. The longitudinal non-parametric analysis for the small sample size was performed with the function Ld.F1. This function performed several tests for the relative treatment effect alternatives for the longitudinal design with one sub-plot factor variable (time in months in this case). Findings were considered statistically significant at 0.05.

## 3. Results

A total of 12 implants were placed in five patients. Three out of five patients were treated in maxillary sites and two in mandibular sites. The post-operative healing in the following 8–9 months was regular for all patients, obtaining good clinical results from a clinical point of view that allowed a subsequent implant placement. In one case there was a limited exposure of the membrane that did not compromise the integration process of the graft and did not impair the regeneration. The exposure was treated with dressings and rinses with a chlorhexidine-based (0.2%) mouthwash. The premature removal of the titanium mesh after exposure was not necessary as it did not compromise the clinical outcome of the bone regenerative procedure. All implants successfully survived and marginal bone levels appeared stable.

**Figure 21:**
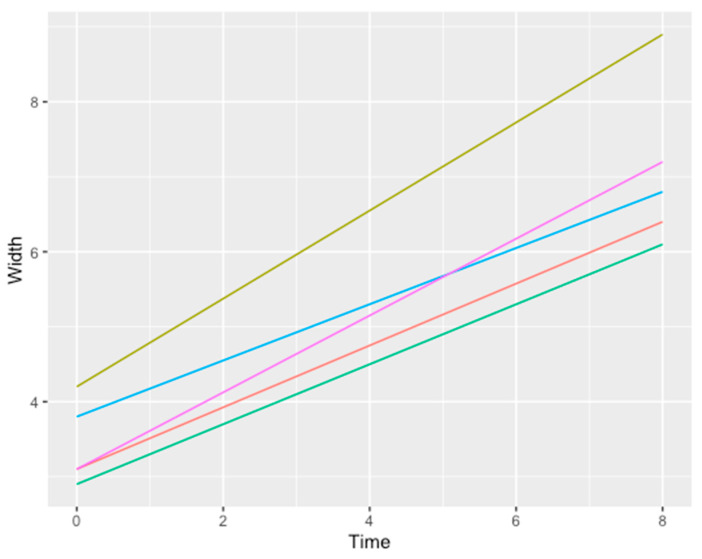
Line chart showing the relation between time measured in months (x axis) and width measured in mm (y axis). Each line represents a different patient. Color legend concerning the data/patients shown in Table 1: grass green (1), blue (3), purple (4), orange (5), bright green (2).

**Figure 22:**
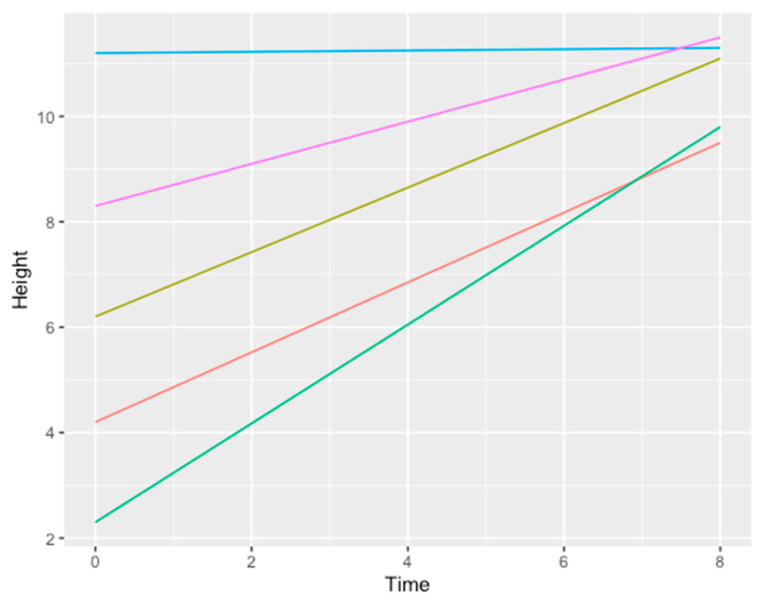
Line chart showing the relation between time measured in months (x axis) and height measured in mm (y axis). Each line represents a different patient gain. Color legend concerning the data/patients shown in Table 1: grass green (1), blue (3), purple (4), orange (5), bright green (2).

From a radiographic point of view (CBTC), satisfactory results were reached both in horizontal and vertical defects (Table 1):

- An average horizontal gain of 3.6 mm ± 0.8 mm (Figure 21)
- An average vertical gain of 5.2 mm ± 1.1 mm (Figure 22)

The Ld.F1 function modeled on width- time gave a significant *p* value of <0.05. The same outcome was found for the height-time relation.

## 4. Discussion

The present case series has shown that the digital workflow for the manufacturing of customized titanium mesh is a safe and efficacious procedure for achieving bone regeneration within large alveolar defects.

The regeneration of bone defects remains a challenging surgical procedure especially in the case of huge bone atrophies. The morphology, quantity and quality of residual bone are important factors to be considered when choosing a reconstructive technique. In any case, reconstructive surgery can be performed using either bone blocks or particulate bone [21,22,23,24,25,26,27] in combination with various types of meshes. Both materials have strengths and weaknesses.

Over the years, scientific research has tried to develop a technique that could overcome the limits of blocks and bone particulate by combining the strengths of both methodologies [28,29].

The use of expanded polytetrafluoroethylene (e-PTFE) meshes in association with particulate bone has been widely described to correct both horizontal and vertical defects. It has been reported to reach between 3.8 mm and 5 mm in thickness increase and 4.1 mm to 5.8 mm in vertical gain [30,31]. However, the GBR technique has been associated with a low level of reproducibility and inadequacy in treating extensive defects, with a high level of complications of up to 15%. The exposure of the membrane and/or subsequent over-infection and loss of part or all of the graft are not infrequent findings [31].

The use of resorbable membranes may reduce the rate of complications but it seems less effective in terms of bone augmentation (average vertical gain = 2.9 mm; average horizontal gain = 3.8 mm) due to the collapse of the membrane within the defect [32].

The GBR technique with a titanium mesh is a regenerative procedure viable in extensive defects with complex morphology. This technique provides great advantages, which derive from the combination of the physical characteristics of the titanium mesh with the physical and histological characteristics of the particulate bone, allowing both good vertical gains (5.8 mm) and horizontal gains (4.6 mm). However, this method is not free of complications (21%) [33].

The realization of customized 3D meshes with CAD-CAM techniques after a careful virtual planned design can favor the treatment of very large defects and also reduce surgical times because the customized grid immediately and perfectly adapts to the site to be rebuilt without the necessity of intra-operative modeling [33,34]. Therefore, CBR avoids all those procedures of cutting, shaping and adapting the old titanium membranes (time-consuming operations), eliminating even the sharp edges created during the modeling of conventional grids that created an irritation of the mucosa, gingival dehiscence and early exposure of the meshes with the risk of failure of the regenerative procedure [35]. The most important factor that can affect the quantity and quality of the regeneration seems to be the exposure of the grid in terms of extension and the timing of appearance although this complication does not seem to compromise the final result [36]. The results of the present case series confirmed this hypothesis. Even though one patient showed a mesh exposure, premature mesh removal was not necessary as it showed no symptoms or signs of superinfection and it was easily recovered. The exposure of the mesh therefore did not affect the final outcome of the regeneration.

Soft tissue management represents the most critical point of this technique, which goes from the design of the surgical flap to the search for a first intention closure without tension. The extension of the incision is also very important: it must be wide, involving two teeth both in the mesial direction and in the distal direction in order to close the defect hermetically and predictably.

The formation of a fibrous granulation tissue below the grid might explain the low rate of infection. This tissue, similar to the periosteum, protects the graft from bacterial contamination and the consequent resorption even in cases of extensive exposure. The real nature and origin of this tissue is not well known [37,38,39,40].

## 5. Conclusions

Within the limits of this study, the results showed that this new technique of personalized bone regeneration (CBR) with the use of custom 3D titanium meshes manufactured with CAD-CAM technology represents a safe, encouraging and predictable alternative for the regeneration of various bone defects. The design of the flap and the closure by primary intention, avoiding the presence of potentially harmful tensions, remain the most critical phases of this custom surgical digitally planned technique. Partial exposure of the mesh does not necessarily lead to the failure of the regenerative technique. All of the authors agree on the decreased incidence of exposure associated with the “poncho” incision compared with the crestal incision. This surgical “poncho” approach could be considered as a favorable and protective factor for the underlying grafted area. In addition, the digital flow and specifically the preventive CBR surgical planning seem to contribute towards making the results encouraging by reducing intraoperative critical issues.

## Figures and Tables

**Table 1:** Bi-dimensional bone gain for each patient.

Patient	Atrophic Site	Initial Width	Initial Height	Final Width	Final Height	Fixture
1	36	4.2 ± 0.8	6.2 ± 0.8	8.9 ± 1.2	11.1 ± 2.2	1
2	24–25–26	2.9 ± 0.9	2.3 ± 1.3	6.1 ± 0.5	9.8 ± 0.3	3
3	12–11–21–22	3.8 ± 0.5	11.2 ± 0.5	6.8 ± 0.3	11.3 ± 0.5	4
4	36	3.1 ± 0.2	8.3 ± 0.8	7.2 ± 0.9	11.5 ± 0.3	1
5	14–15–16	3.1 ± 0.5	4.2 ± 0.2	6.4 ± 0.5	9.5 ± 0.5	3
